# Assessing Neuromuscular Adaptation and Kinetic Symmetry in Elite Para-Kayakers: A Wearable Body Sensor Network Case Series

**DOI:** 10.3390/s26165018

**Published:** 2026-08-07

**Authors:** Giovanni Saggio, Leonardo Pierucci, Luca Pietrosanti

**Affiliations:** Department of Electronic Engineering, University of Rome Tor Vergata, Via Politecnico 1, 00133 Rome, Italy

**Keywords:** motion analysis, adaptive symmetry, athlete impairment, wearable sensors, body sensor network

## Abstract

**Highlights:**

**What are the main findings?**

- A wearable 13-node wireless body sensor network (BSN) successfully quantified three-dimensional segment orientation trajectories in elite para-kayakers, proving highly feasible for capturing granular, dynamic biomechanical profiles.
- Bilateral upper-limb kinematic symmetry does not directly guarantee superior mechanical efficiency or propulsive power; removing a prosthetic limb improved trajectory symmetry in one KL3 athlete but triggered a severe 98 W (45.8%) decrease in average power output.

**What are the implications of the main findings?**

- The newly formulated ROM Ratio acts as a superior diagnostic kinematic coefficient by preserving both the magnitude and polar direction (left-to-right dominance bias) of contralateral joint asymmetries, proving highly effective for real-time, on-field coaching.
- Equipment profiling in para-sports should prioritize custom orthopedic boundary conditions (bespoke seating and footrests) over generic, group-averaged symmetry targets to maximize individual force transmission along the altered kinetic chain.

**Abstract:**

Movement analysis is essential for high-level athletes to perform at their best. Such analysis necessarily involves studying the characteristics of one’s own body, whose adaptive processes are crucial for understanding how each athlete responds to sudden changes. In this preliminary exploratory work, the paddling techniques of a limited, heterogeneous cohort of eight athletes were evaluated: four who were non-impaired and four who showed different classes of impairment (two KL3, one KL2, and one KL1). Each non-impaired athlete underwent three test series—one with a footrest, one without a footrest, and another with a modified footrest. KL3 athletes performed two tests, one with the prosthetic leg and one without, while KL2 and KL1 athletes completed a standard sprint. Measurements were obtained using a set of inertial sensors. The results showed a tendency toward greater symmetry in shoulder trajectories when the footrest was removed, and athletes improved their performance when sprinting under a modified configuration. These findings provide insight into how the body compensates for the absence of leg support, including changes in muscle recruitment and a tendency toward the better control of overall movement. Given the inherent constraints of the small sample size (n = 8), these results demonstrate the potential of IMU networks for personalized biomechanical assessment, providing descriptive, case-level insights rather than statistically generalizable trends.

## 1. Introduction

Flat-water kayaking is a highly demanding discipline in which athletic performance is dictated by the attainment of optimal velocity and peak power output, achieved through the integration of distinct biomechanical patterns—a principle applicable across diverse sporting contexts [1]. From a biomechanical perspective, the paddling cycle represents a rhythmic interaction between the athlete, the paddle, and the fluid environment, governed by the equilibrium between propulsive forces and hydrodynamic drag [2]. This cycle is categorized into three primary phases: the catch, the pull (characterized by blade immersion and propulsion generation), and the recovery or ‘air’ phase [3,4]. During the pull phase, the athlete must overcome active drag, primarily wave-induced, which increases non-linearly with velocity. Conversely, the recovery phase is marked by a period of a total absence of propulsion as both limbs are outside the water to prepare for the subsequent stroke [5]. This phase refers to the brief transitional interval between the exit of one blade and the catch of the contralateral one. During this specific window, both blades are simultaneously out of the water to facilitate maximal torso rotation and optimal kinetic chain loading; however, it is also the interval where hydrodynamic drag acts as a decelerating force on the hull [2].

Consequently, the kayak does not experience continuous propulsion; rather, it undergoes a series of cyclic accelerations followed by brief periods of deceleration. The duration of this non-propulsive gap is inversely related to paddling frequency: it is minimized during high-cadence 200 m sprints but becomes more pronounced in longer distances, where efficiency and rhythmic consistency are prioritized.

Furthermore, while the submerged blade serves as a stabilization point, its absence during the recovery phase significantly reduces system stability, necessitating complex neuromuscular adjustments—specifically involving the core and lateral abdominal musculature—to maintain equilibrium [6,7]. Standard racing boats (5.20 m length, 12 kg mass) are characterized by a narrow hull geometry designed for minimal resistance. Given the absence of inherent lateral stability, the athlete’s postural dynamics are critical for stability and propulsion. Furthermore, technical requirements vary by event duration: 200 m and 500 m sprints necessitate explosive power outputs, while longer distances (1000–2000 m) require a focus on stroke efficiency and rhythmic consistency [8].

The biomechanics of kayaking involve a closed kinetic chain starting from the feet and progressing through the pelvis and trunk to the paddle [9]. However, in para-kayaking, anatomical and functional impairments alter this chain. Para-athletes are classified based on their functional capacity into KL1, KL2, and KL3 categories [10]. KL1 participants lack trunk and leg function, necessitating a primary reliance on the upper body. KL2 athletes retain partial trunk and leg control, though their capacity for force transmission remains suboptimal. In contrast, KL3 athletes maintain full trunk stability and partial lower-limb involvement, allowing for a more complete closure of the kinetic chain compared to the more impaired classes.

Characterizing the impact of specific physical impairments on biomechanical adaptations and symmetry is critical for tailoring coaching interventions and refining equipment design. Although optical-based motion capture systems remain the gold standard [11,12], their deployment is frequently constrained by the challenges of the aquatic environment and their limited capacity to monitor the highly individualized compensatory mechanisms inherent to para-kayaking [13].

Dry-land ergometry offers a viable alternative to field-based aquatic testing [14]. By utilizing a pulley-and-cable system connected to the shaft, the ergometer approximates hydrodynamic resistance, allowing for a partial replication of the stroke kinematics. Dry-land indoor ergometry presents distinct hydrodynamic boundaries compared to real-water propulsion; however, high-fidelity kayak simulators (equipped with mobile seat trolleys and airbrake flywheels) have been extensively validated to recreate upper-body biomechanics and kinetic coordinate transfer [15]. By isolating the closed kinetic chain, this indoor setup eliminates stochastic environmental confounding factors—such as wind shear and ambient water drag—to provide a repeatable, high-precision laboratory baseline crucial for detecting granular musculoskeletal adaptation thresholds [16]. Additionally, high-fidelity ergometers provide real-time feedback on key kinematic and kinetic parameters, such as instantaneous power, pace, and interval completion times [17].

The practical utility of wearable devices aligns with the requirements for in situ kinematic data acquisition, offering an alternative to laboratory-grade optical motion capture [11,18]. Despite their high precision, optoelectronic systems present several logistical challenges, such as high capital expenditure and the requirement for expert operation. To address these constraints, recent developments have pivoted toward ego-centric sensing paradigms. Unlike traditional optical setups—which require external sensors to track markers on the subject—these emerging technologies utilize on-body sensors that directly monitor moving elements [13,19].

Within this framework, various methodologies for kinematic and kinetic analysis in kayaking have been documented. For instance, in [20], the authors utilized strain gauges and force-sensitive resistors to quantify paddle deflection and the center of pressure (CoP) exerted at the footrest. Similarly, handgrip pressure on the paddle shaft has been recorded using piezoresistive sensor matrices [21], while fiber Bragg grating arrays have been employed as compression load sensors to map blade loading distributions [14]. Other investigations have focused on temporal and resistive parameters; in [9,22], force sensors were employed to assess paddle immersion depth, pull-phase duration, and stroke force. In terms of cadence monitoring, ref. [23] leveraged paddle-mounted accelerometers to determine strokes per minute. More comprehensive approaches, such as those in [24,25], have integrated Inertial Measurement Units (IMUs) with Global Positioning Systems (GPSs) and force transducers to capture a full suite of kinematic (frequency, displacement, and orientation: roll, pitch, yaw) and dynamic (paddle and footrest forces) variables. Distinctively, in [25], the authors also demonstrated the use of body-worn IMU arrays—positioned on the limbs and trunk—to reconstruct joint angles during the paddling cycle.

IMUs represent a cornerstone in human-centric kinematic monitoring, providing high-fidelity objective data across multiple sectors. Beyond clinical and gait analysis [26,27], these sensors are integral to activity recognition [28,29], human–robot/computer interaction [30,31], ergonomic risk assessment [32], and teleoperation [33,34]. Recently, their application has expanded significantly into sports science, facilitating the granular analysis of athletic performance [35,36].

Building upon prior research, this pilot study evaluates the feasibility of an IMU-based body sensor network to characterize individual adaptive mechanisms in both able-bodied and para-athletes. Specifically, it examines how biomechanical techniques shift under varied boundary conditions during 200 m sprint trials. The implementation of the ROM Ratio—a novel kinematic index—further demonstrates the capacity of wearable sensors to detect granular compensatory nuances that are typically obscured by standardized assessment protocols.

## 2. Materials

### 2.1. Participants

Eight athletes participated, four able-bodied participants (K1 class) and four para-athletes (1 KL1, 1 KL2, and 2 KL3 classes), classified according to Paracanoe Kayak guidelines [10]. Impairments included complete spinal lesion (KL1), partial spinal lesion (KL2), and unilateral lower-limb amputations (KL3). This diversity in profiles was essential to highlight different motor compensation strategies for optimal performance results. Athletes’ characteristics are shown in Table 1, with a mean age of 24 ± 3 years.

### 2.2. Ergometer

All the athletes performed sprint trials using a Dansprint PRO Kayak Ergometer (by Dansprint ApS, Herlev, Denmark) [37,38], with the related software Dansprint Analyser (v.1.11), that features adjustable components, such as a mobile trolley, an ad hoc seat, and a carbon bar with ropes connected to an airbrake flywheel to mimic natural paddle movement and provides real-time training data on a touch screen display.

### 2.3. Body Sensor Network (BAN)

The body sensor network comprised 13 CE-certified Movit G1 IMUs (Captiks Srl, Rome, Italy), which have been previously validated [18] and successfully employed as effective tools for human kinematic assessment [26,39,40]. Data acquisition and processing were performed using the associated Motion Studio 3D software (v2.5.003). The sensor modules (48 × 39 × 18 mm, 25 g) feature a triaxial accelerometer (±16 g max, set to ±2 g), a triaxial gyroscope (±2000 deg/s full scale), and a Microprocessor Unit (MPU model 9250, InvenSense, San Jose, CA, USA). Acquisition was performed at a sampling rate of 200 Hz, with wireless data transmission via a high-throughput, low-latency (system delay < 15 ms) 2.4 GHz IEEE 802.15.4 [41] wireless protocol with real-time packet loss buffering.

## 3. Methods

### 3.1. BAN Calibration and Placement

**Figure 1:**
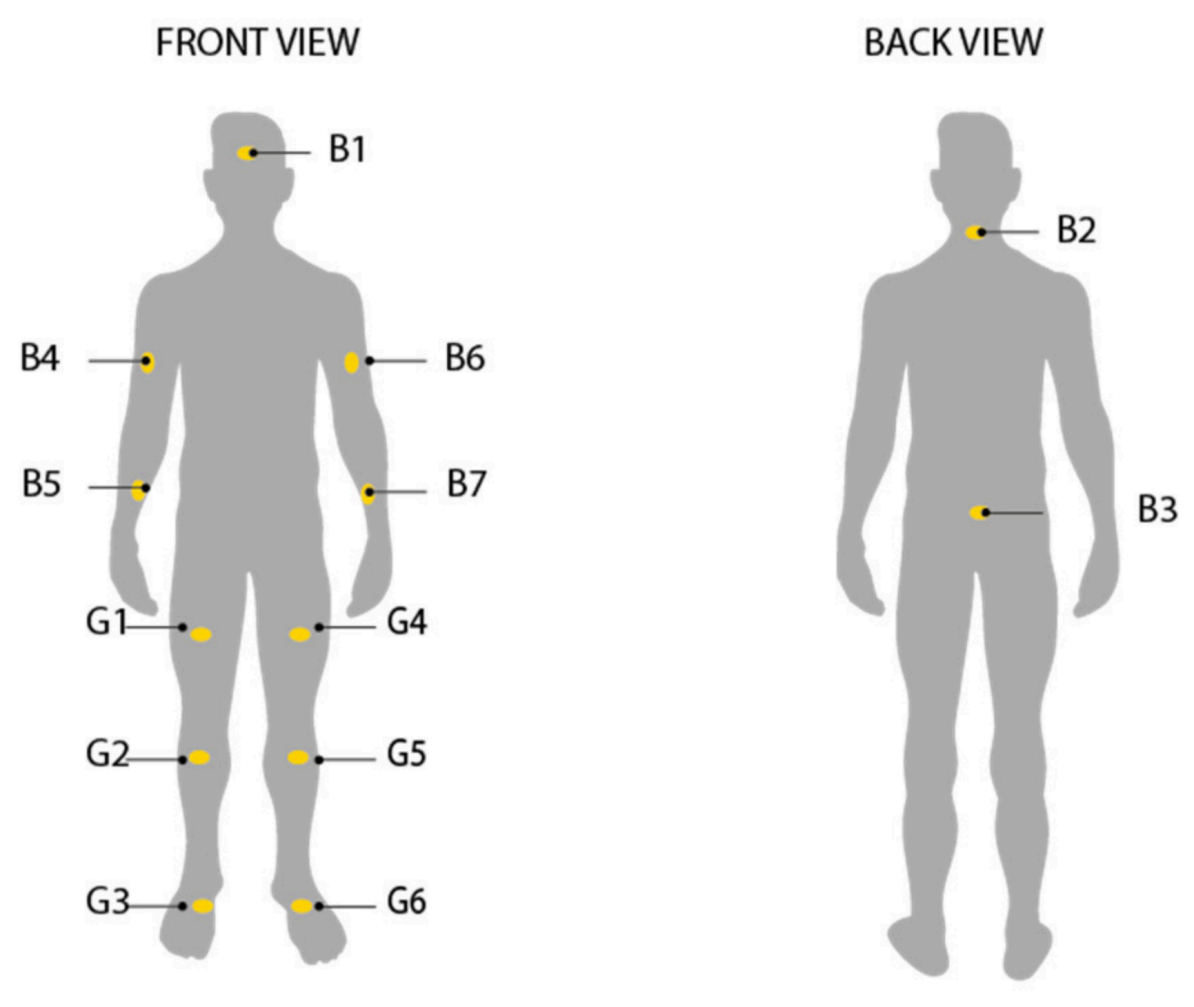
A total of 13 IMUs are located on body segments: B1, 4, 5, 6, and 7 on the upper body; G1, 2, 3, 4, 5, and 6 on the lower body; B2 and 3 on the back.

To establish anatomical correspondence, local sensor frames were aligned with respective segment coordinate axes using a patented docking and calibration procedure (no. 102016000041519). Prior to testing, a static standing calibration posture mapped the technical sensor frames to the standardized anatomical coordinate system, compensating for potential strapping misalignments. Seven sensors were placed on the upper body and six on the lower body using Velcro straps (Figure 1).

B1, B2 and B3 form an imaginary vertical line that will represent the head and the torso. B1 is placed on the forehead, B2 is placed just below the c7 vertebra and B3 is placed in the back lumbar zone. For the arms, B4 and B5 (and B6 and B7) are placed just below the deltoid and at the center of the forearm respectively. With this configuration, the sensors remained firm in place without slipping or loosening. For the legs, the sensors G1, G2 and G3 (and G4, G5 and G6) were placed at the mid-thigh, just below the knee and the forefoot respectively.

For K1 and KL3 athletes, the whole set of 13 sensors was used, 7 placed on the upper body (B1 to B7) and 6 on the lower body (G1 to G6). For KL2 and KL1 athletes, only B1 to B7 were placed on the upper body.

### 3.2. Motor Test Protocol

**Figure 2:**
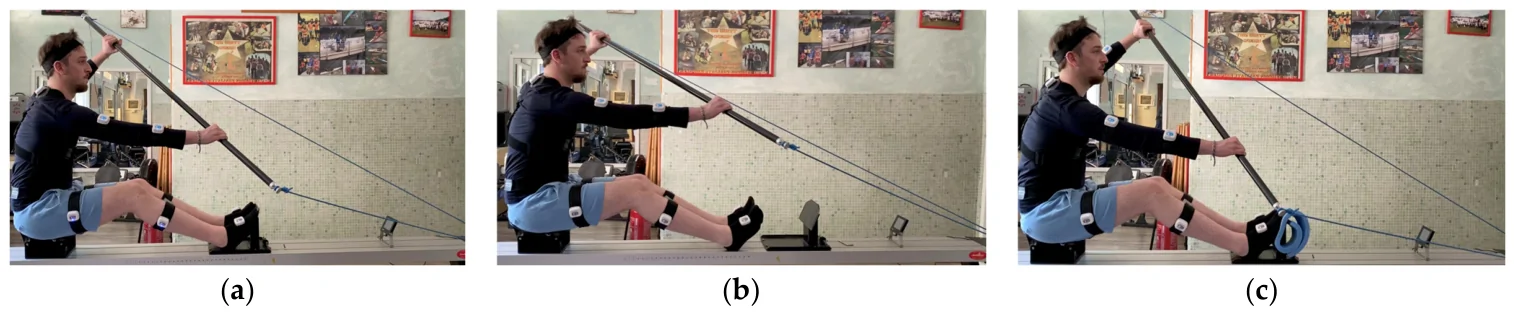
(**a**) Normal configuration. (**b**) Configuration without footrest. (**c**) Modified configuration.

By implementing a protocol co-designed with elite coaches, this exploratory study investigates the viability of tailored sensor-based adjustments for individual athletic profiles focused on:

- KL1 and KL2: One sprint under competition conditions;
- KL3: Two sprints, one with and one without the prosthesis;
- K1: Three sprints, one with full leg use, one without a footrest, and one with a modified footrest disposition (Figure 2).

For KL2, the legs were blocked with straps, and for KL1, the legs were blocked with straps combined with a plastic ad hoc leg bracket.

KL3 athletes performed two sprints in the same session, with a 40 min interval between trials.

K1 athletes performed the first two sprints (normal conditions and without footrest) in the same session, with a 40 min interval between the trials. The third sprint was performed the following day.

**Figure 3:**
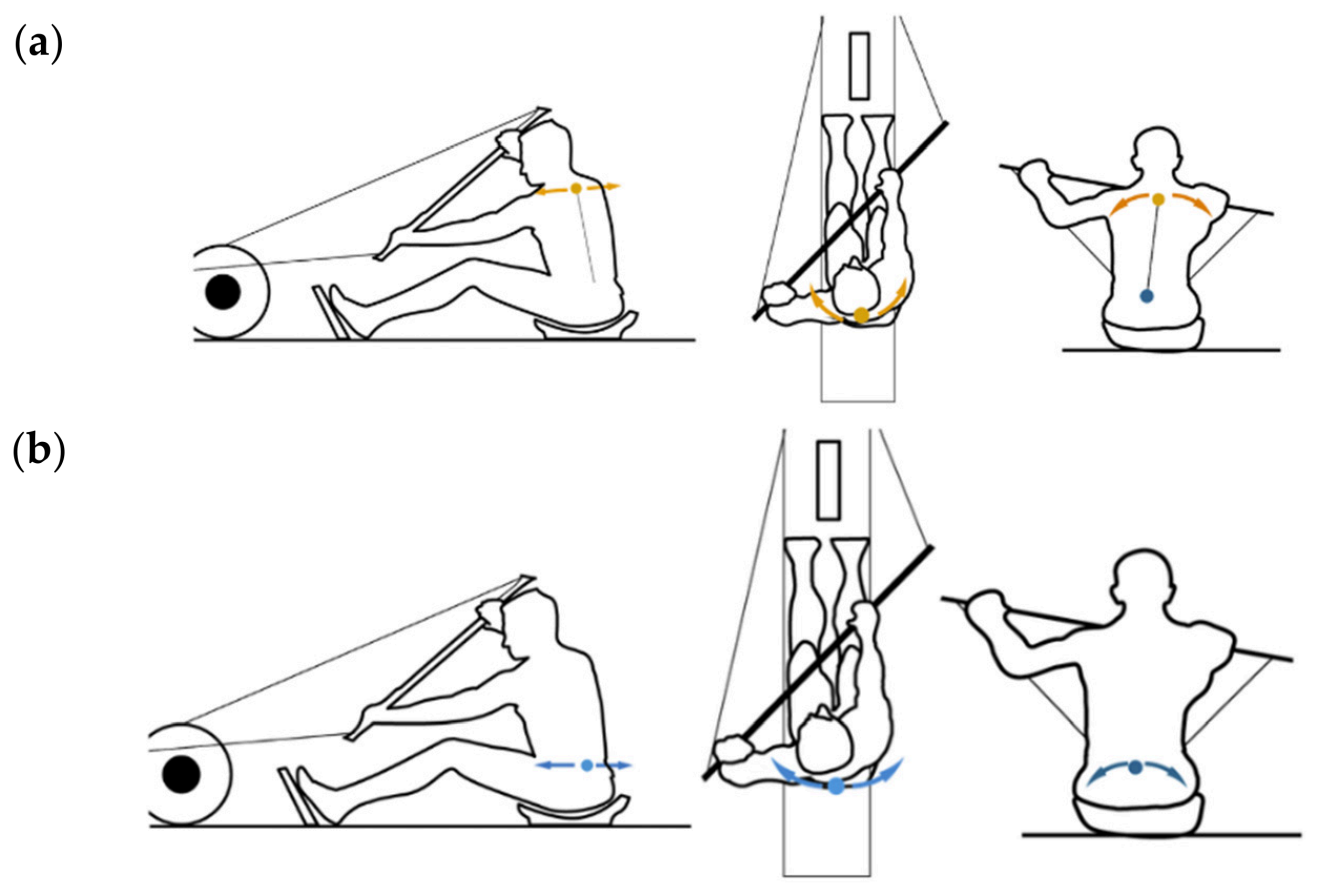

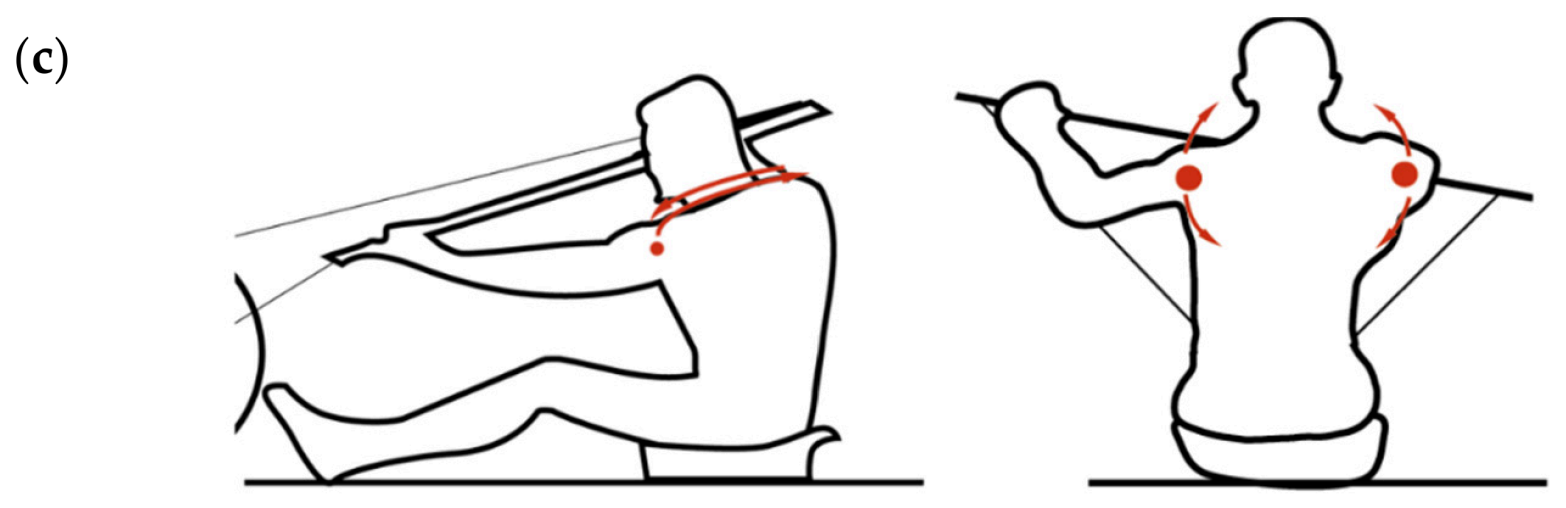
(**a**) Spine flexion–extension, rotation and lateral bending; (**b**) pelvis tilt, rotation and lateral bending; (**c**) shoulder flexion–extension, abduction–adduction.

Prior to the third sprint, K1 athletes completed a 30 s paddling recording at a controlled, submaximal pace. This recording established a kinematic baseline to identify potential asymmetries in trunk and pelvic oscillations before the primary sprint trials (Figure 3).

The footrest setup was systematically modified to address the identified asymmetries. Due to a lack of standardized quantitative guidelines for para-kayak footrest optimization, a user-centered methodology was employed. Adjustments—including polystyrene blocks and heel supports—were tailored to each athlete’s self-reported ergonomic comfort and stability. The relationship between structural footrest changes and improved lateral symmetry is modulated by complex somatic adaptation kinetics. Rather than establishing an immediate linear correlation, the motor control system exhibits an active accommodation period (joint repatterning latency), during which the central nervous system integrates novel somatosensory and mechanoreceptive feedback to optimize force transmission dynamic patterns. For instance, consider an athlete whose pelvis is rotated towards the left (namely, with that body portion facing left). In this case, it is necessary to place an object that increases the thickness on the right side of the footrest to encourage the athlete to push against the right side, thereby compensating for the asymmetry.

Four controlled and submaximal paddling sessions were conducted to evaluate how targeted configuration changes affected symmetry. Adjustments focused on the rotational and lateral bending components of the spine and pelvis (Figure 3). Although zero-value oscillations are unattainable due to physiological variability, we pursued ‘adaptive symmetry’ to enhance performance. Because effective kayaking techniques require specific ranges of spinal and pelvic flexion and tilting, maintaining these oscillations within functional limits is more critical than their elimination.

During these controlled paddling sessions, we obtained the values of the aforementioned parameters (in degrees) from the IMUs using a custom MATLAB (v.2024b, The MathWorks, Inc., Natick, MA, USA) script. These real-time orientation metrics served as a reference for the systematic, iterative adjustment of the footrest configuration (e.g., through the addition of tailored supports) until the desired setup was reached, namely a condition in which the chosen parameters (lateral bending and rotation of the spine and pelvis) approached 0 ± 2° (negative leftward, positive rightward). Once the setup was optimized, each athlete completed a 200 m sprint with the personalized configuration, as shown in Table 2.

### 3.3. Data Processing and ROM Ratio

Data were processed in real time using Motion Studio 3D analyzer software (v2.5.003, Captiks Srl, Rome, Italy), yielding three-dimensional (3D) orientation coordinates represented as unit quaternions. These quaternions were subsequently transformed into joint Euler angles representing anatomical angular displacements. The following were evaluated with a MATLAB custom script (v.2024b):

- Spine: Flexion–extension, lateral bending, rotation;
- Pelvis: Tilting, lateral bending, rotation;
- Shoulders: Bilateral flexion–extension, abduction–adduction.

These parameters were selected based on the findings of previous research [6,20,21] and on specific requirements made by professional coaches involved in this study.

Soft tissue artifact (STA) propagation and sensor-to-skin micromovements were minimized by employing high-tension elastic coupling straps. Because the angular ranges of motion of the trunk and upper-limb segments during paddling (typically >60°) are significantly larger than the maximum translational sliding of the sensors over the skin (generally <5 mm), the signal-to-noise ratio remained exceedingly high. The computational mitigation of high-frequency micromovement noise was successfully achieved via the a posteriori digital filtering of kinematic data with a zero-phase, 4th-order low-pass Butterworth filter (cut-off frequency of 6 Hz) [42], effectively smoothing high-frequency noise without introducing phase distortion.

Owing to the pilot nature of this study and the broad functional spectrum of the cohort, which included both able-bodied and KL1 athletes, we adopted a descriptive analytical framework. This methodology prioritizes the sport-specific significance of kinematic profiles and idiosyncratic compensatory strategies, focusing on individualized optimization for coaching and equipment rather than statistical generalizability.

Moreover, we introduced the ROM (Range Of Motion) Ratio as a new empirical parameter to quantify asymmetry, defined as the ratio between the left shoulder’s flexion–extension ROM (deg.) and right shoulder’s flexion–extension ROM (deg.). ROM Ratio values approaching 1 indicate greater symmetry, though this does not inherently guarantee superior performance. The newly formulated ROM Ratio acts as an intuitive, unitless kinematic coefficient that preserves both the magnitude and polar direction (left-to-right dominance bias) of contralateral shoulder flexion–extension. Unlike the already adopted Symmetry Index (SI) [43,44], which compresses asymmetric variances into absolute scale percentages, the ROM Ratio directly projects the functional biomechanical asymmetry of the upper body, rendering it highly accessible for real-time coach–athlete communication during dynamic trials. Typically, a larger absolute Range of Motion (ROM) is preferable in this sport, as maximum limb extension is critical for maximizing water displacement and stroke length. However, while a substantial ROM is a functional prerequisite, it is not sufficient for high-level performance unless it is balanced by optimal bilateral symmetry, as quantified by a ROM Ratio approaching 1.0.

## 4. Results

Due to the pilot nature of this investigation and the heterogeneous functional profiles of the participants, the following results are presented as descriptive, case-level observations. No inferential statistical testing (e.g., paired comparisons or confidence intervals) was performed for within-athlete condition comparisons. Consequently, these findings should be interpreted as individualized biomechanical profiles and diagnostic indicators rather than statistically significant trends applicable to the broader para-kayaking population.

Table 3 reports trial data for every athlete, classified according to their impairment’s category, time (s), average power (W), and ROM Ratio.

For K1 athletes, trial #1 consists of sprints in normal conditions; trial #2 consists of sprints without a footrest; trial #3 consists of sprints with a modified disposition. For KL3 athletes, trial #1 consists of sprints with a prosthesis; trial #2 consists of sprints without a prosthesis. For KL1 and KL2, only trial #1 sprinting was possible.

**Figure 4:**
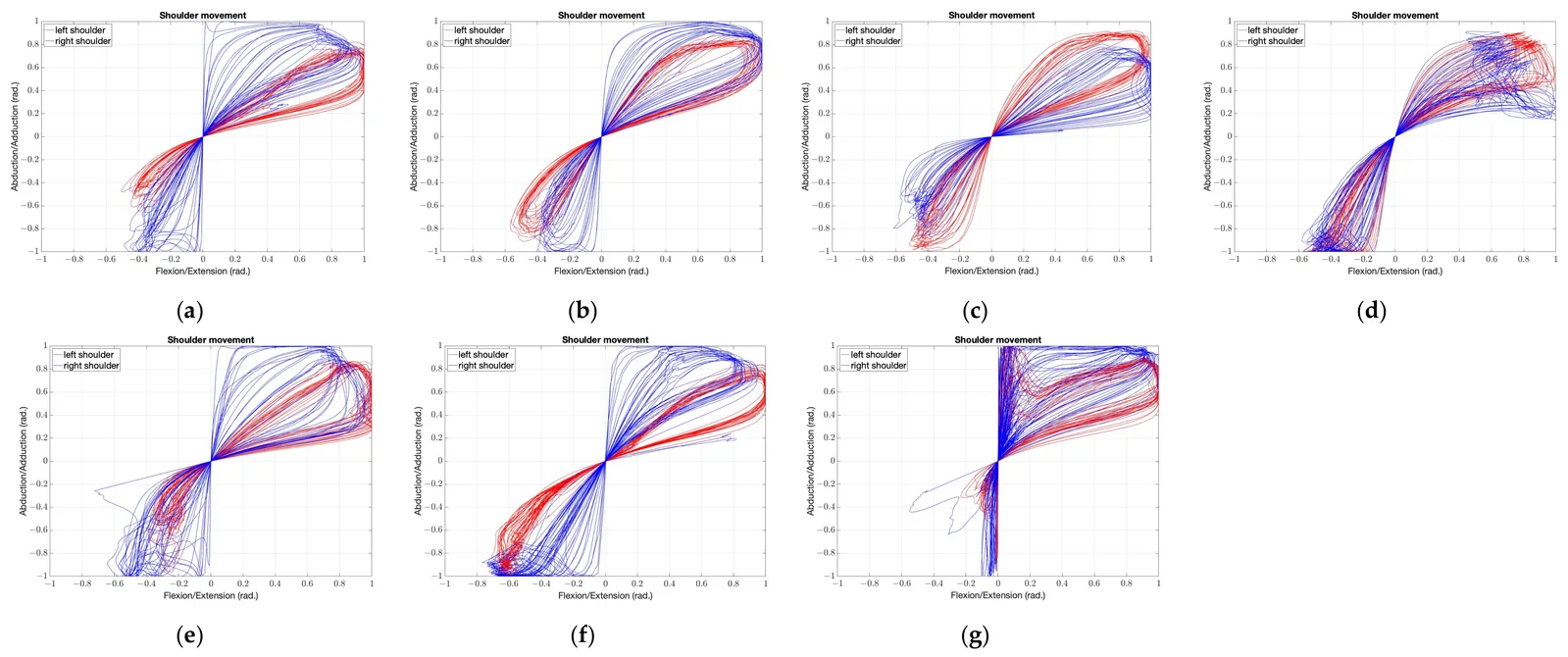
Shoulder flexion–extension (x-axis) and abduction–adduction (y-axis), in radians, related to K1 (**a**) normal conditions, (**b**) without footrest, (**c**) modified disposition; KL3 (**d**) with prosthetic limb, (**e**) without prosthetic limb; (**f**) KL2, (**g**) KL1.

Figure 4 depicts shoulder movements, as recorded by the sensors from B1 to B7 (Figure 1), during the whole sprinting sessions across categories. These trajectories illustrate combined horizontal (flexion–extension) and vertical (abduction–adduction) motions as ROM values, since they show us the range of movement realized by the shoulders themselves. In the upper-right quadrant, extension and abduction occur, while in the bottom-left quadrant, flexion and adduction are observed.

## 5. Discussion

The analysis revealed that paddling performance depends on both biomechanical technique and individual physiological conditions. Wearable IMUs enabled a detailed reconstruction of joint trajectories, highlighting asymmetries and supposed compensatory strategies that varied significantly among participants.

It must be noted, however, that since this pilot study did not incorporate direct physiological measurements (e.g., EMG), the mechanistic explanations provided—such as changes in muscle recruitment patterns or increased segment stiffness via co-contractions—should be interpreted as speculative hypotheses derived from kinematic observations rather than confirmed physiological events.

Importantly, performance should be assessed by comparing each athlete’s trials under different conditions rather than across athletes.

The preference for a descriptive framework allowed for a detailed examination of each individual athlete, which is critical for attaining high-level performance. In the context of para-sports, where impairments are highly individualized [36], a case-series descriptive approach can offer more actionable insights for technical development than aggregate data which may fail to capture unique neuromuscular adaptations.

Generally, the results of this exploratory investigation suggest that, for K1 athletes, removing the footrest is associated with an increase in shoulder symmetry, although average power did not improve. This observed pattern may suggest that restricting the use of the legs, which typically serve as the primary support force transmission along the kinetic chain, could potentially lead to a higher engagement of abdominal and pelvic muscles to uphold trunk stability. This compensatory response suggests that limiting lower-limb drive—the standard feedback conduit of the closed kinetic chain—forces the kinetic system to rely extensively on core pelvic–lumbar stabilizers to maintain posture. Lacking a mechanical buttress to discharge reaction forces, the central motor command likely increases segment stiffness through generalized pelvic–abdominal co-contractions to counteract inertial trunk displacement during axial rotation.

Following the framework established in [6,45], the paddling cycle was divided into three distinct phases: catch, pull (at least one blade in the water) and exit/air (both blades in the air). The air phase is often identified as the period of the lowest equilibrium, as the absence of blade–water interaction increases the reliance on internal stability [2]. Thus, the preliminary observations of the trajectories in Figure 3 suggest that flexion–adduction during the air phase appears to be the most sensitive to changes in the experimental setup. From a theoretical standpoint, the kayak undergoes deceleration whenever propulsion is absent, as hydrodynamic drag continuously opposes motion [45]. While the submerged blade provides a propulsive force, it also acts as a stabilization point; therefore, its absence during the air phase may require increased postural control from the athlete to maintain equilibrium. To maintain or increase velocity, the propulsive force must equal or exceed the total drag. Consequently, during the air phase, the lack of propulsion allows hydrodynamic drag (which scales with velocity) to act as a significant braking force on the hull. Furthermore, the trajectories recorded during the flexion–adduction phase seem to show more pronounced variations compared to the extension–abduction phases (upper-right quadrants), which remained relatively consistent across different categories. For instance, a comparison of the (a), (d), and (g) profiles reveals similar extension–abduction patterns in both morphology and magnitude, despite marked differences in the lower-left quadrant. Even within K1 athletes (a, b, and c), while trajectories maintain a characteristic ‘teardrop’ shape, they exhibit notable inter-individual differences in terms of displacement and linearity. This heterogeneity might highlight the critical nature of the air phase: lacking the hydrodynamic support of the blade in the water, the paddling technique becomes more susceptible to individual anatomical constraints and potential neuromuscular adaptations required to preserve vessel stability.

Athlete 3 (K1) exemplified this (Figure 3a–c): sprint time improved from 55 s to 47 s with the modified setup, power increased from 169 W to 178 W, and symmetry increased too (ROM Ratio ~ 0.84).

Regarding the KL3 category (Figure 4d,e), observations from this pilot study suggest that the use of a prosthesis may significantly influence both performance and symmetry, although individual responses varied between the two participants. For athlete #5, starting from his standard condition (49 s, 243 W, ROM Ratio 1.05), removing the prosthesis was associated with a marked decrease in power and a higher ROM Ratio, which might indicate a reduction in both stability and stroke effectiveness (53 s, 121 W, ROM Ratio 1.31). In contrast, athlete #6 showed an improved ROM Ratio (1.08) without the prosthesis; this could potentially suggest a higher degree of individual functional adaptation to the impairment over time. While athlete #6 appeared more symmetrical in the absence of the prosthesis, the substantial drop in power output (ΔW = 98 W) compared to athlete #5 (ΔW = 121 W) highlights that geometric symmetry alone does not necessarily guarantee performance gains, a finding that appears consistent with the literature emphasizing the role of coordinated force generation over pure symmetry [11,46]. These results could support the hypothesis that individual neuromuscular strategies are crucial in compensating for unilateral limb loss, though the limited sample size prevents broader generalizations [45].

The comparison between KL2 (Figure 4f) and KL1 (Figure 4g) further illustrates the potential impact of trunk function on paddling kinematics. The KL2 athlete, possessing partial spinal function and some residual trunk control, maintained relatively coordinated trajectories. Conversely, the KL1 athlete exhibited what appeared to be more variable and less organized trajectories, particularly during the air phase. This observation may suggest that, in the absence of significant trunk control, the athlete prioritizes maintaining balance over propulsive efficiency during the most unstable part of the stroke. These preliminary findings appear to align with previous research regarding the trunk’s essential role in movement coherence in para-sports [6,45].

While the heterogeneous, small-cohort design (n = 8) inherently limits group-level statistical generalizability, this clinical case-series approach demonstrated a structural superiority for elite para-sports science. Given that distinct physical impairments (e.g., complete spinal lesion vs. unilateral amputations) manifest in highly individualized musculoskeletal compensatory pathways, group aggregation would obscure clinical nuances and critical neuromuscular strategies with misleading averages. Therefore, a highly localized, idiographic framework is required to successfully map bespoke adaptation curves for athletic equipment profiling. A key goal is to develop standardized methods to determine optimal seating and disposition for each athlete, tailored to individual anatomy and maximizing power output. The lack of direct physiological measurements, such as EMG, means that the discussed muscle recruitment patterns remain speculative hypotheses that require further validation.

## 6. Conclusions

This preliminary pilot study suggests that functional control and adaptive compensation are key factors to consider in kayaking performance, particularly within the diverse landscape of para-sports. Our preliminary findings indicate that strict bilateral symmetry may not be the sole determinant of efficiency; rather, the ability of the athlete to adapt to different boundary conditions (such as changes in footrest setup or the use of prostheses) may play a significant role. In this small cohort, athletes with greater residual trunk function or stable lower-limb support seemed to maintain more coordinated movement patterns, whereas those with more significant impairments tended to exhibit higher variability, potentially prioritizing stability over pure propulsive power. However, due to the small and heterogeneous nature of the sample, these findings must be interpreted as descriptive case-series observations. While these results indicate the feasibility of utilizing IMU-based body sensor networks for individual profiling, they do not allow for broader group-level generalizations regarding the para-kayaking population, which could be a potential topic for future studies.

## Figures and Tables

**Table 1:** Athletes’ height, mass, sex, category, and impairment. For athletes #7 and #8, height and mass were not collected due to the functional constraints of the wheelchair. Athletes 5 and 6 had a removable prosthetic limb.

Category	Athlete #	Height (cm)	Mass (kg)	Sex	Impairment
K1	1	172	61	F	None
	2	168	54	F	None
	3	175	60	M	None
	4	188	80	M	None
KL3	5	183	84	M	Left trans-tibial amputation
	6	179	87	M	Right trans-femoral amputation
KL2	7	-	-	M	Partial spinal lesion D12-L1
KL1	8	-	-	F	Complete spinal lesion D12-L1

**Table 2:** Kinematic asymmetry and footrest modification for K1 athletes.

K1 Athlete #	Identified Kinematic Asymmetry	Footrest Modification
1	Rightward spine rotation	Polystyrene block between the left foot and the footrest
2	Spine lateral bending to the left side	Polystyrene block support below the left heel
3	Leftward pelvic rotation	Polystyrene block between the right foot and the footrest
4	Leftward pelvic rotation	Polystyrene block between the right foot and the footrest

**Table 3:** Measured data, in terms of time (rounded to whole, sec), average power (W), and ROM Ratio, that resulted from the trials of each athlete.

Category	Athlete #	Trial #	Time (s)	Average Power	ROM Ratio
K1	1	1	53	168	0.88
		2	61	114	0.77
		3	54	163	0.90
	2	1	61	112	0.79
		2	69	76	1.02
		3	61	109	1.17
	3	1	55	169	1.25
		2	71	79	0.99
		3	47	178	0.84
	4	1	52	216	1.47
		2	68	96	1.23
		3	52	232	0.88
KL3	5	1	49	243	1.05
		2	53	121	1.31
	6	1	50	214	1.27
		2	54	116	1.08
KL2	7	1	48	230	1.27
KL1	8	1	64	99	1.49

## Data Availability

Data are available upon reasonable request.

## Abbreviations

The following abbreviations are used in this manuscript:

KL1	Para-athlete with no or very limited trunk function and no leg function
KL2	Para-athlete with partial trunk and leg function
KL3	Para-athlete with full trunk function and partial leg function
K1	Able-bodied athlete (kayak single)
IMU	Inertial measurement unit
GPS	Global positioning system
MPU	Microprocessor unit
STA	Soft tissue artifact
ROM	Range of motion
